# Tgm2‐Catalyzed Covalent Cross‐Linking of IκBα Drives NF‐κB Nuclear Translocation to Promote SASP in Senescent Microglia

**DOI:** 10.1111/acel.14463

**Published:** 2025-01-03

**Authors:** Zhiqiang Li, Tianxiang Wang, Sijing Du, Zelong Miao, Yujiao Zhao, Yuxiang Tang, Xianbin Meng, Shangcheng Yu, Dongyuan Zhang, Hao Jiang, Kunlin Du, Wei Wei, Haiteng Deng

**Affiliations:** ^1^ MOE Key Laboratory of Bioinformatics, Center for Synthetic and Systematic Biology, School of Life Sciences Tsinghua University Beijing People's Republic of China; ^2^ Wangjing Hospital, China Academy of Chinese Medical Sciences Beijing People's Republic of China

**Keywords:** cross‐linking, IκBα, NF‐κB, SASP, senescent microglia, senomorphics, Tgm2

## Abstract

Microglia, as resident immune cells in the central nervous system (CNS), play a crucial role in maintaining homeostasis and phagocytosing metabolic waste in the brain. Senescent microglia exhibit decreased phagocytic capacity and increased neuroinflammation through senescence‐associated secretory phenotype (SASP). This process contributes to the development of various neurodegenerative diseases, including Alzheimer's disease (AD). In this study, we found that SASP was elevated in senescent microglia, and proteomics showed that Tgm2 was upregulated. Mechanistically, we revealed that Tgm2‐catalyzed covalent cross‐linking of IκBα at K22 and Q248 residues in the cytoplasm of microglia, resulting in the reduction of IκBα and nuclear translocation of NF‐κB to promote SASP production. Treatment of senescent microglia with Tgm2 inhibitors (Tg2‐IN1 and Cys‐D) resulted in reduced NF‐κB nuclear translocation and decreased SASP. Additionally, oral administration of Cys‐D significantly improved the aging phenotype in aged mice. To summarize, Tgm2 is a potential target for antiaging, and inhibitors of Tgm2 can serve as novel prophylactics or senomorphics.

## Graphical abstract

Tgm2 is highly expressed in senescent microglia. The Tgm2‐NF‐κB‐SASP loop facilitates senescence in microglia. Tgm2‐catalyzed covalent crosslinking of IκBα at K22 and Q248 in the cytoplasm of senescent microglia leads to a reduction in IκBα levels and NF‐κB nuclear translocation. Cys‐D effectively disrupts the Tgm2‐NF‐κB‐SASP loop and ameliorates age‐relevant phenotypes in aged mice.

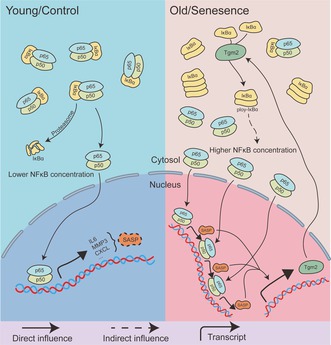

**Abbreviations**
AD: Alzheimer's disease
CNS: central nervous system
Cys‐D or CD: cystamine dihydrochloride
ETOP: etoposide
SASP: senescence‐associated secretory phenotype
Tg2‐IN1: Tgm2 inhibitor, CAS no: 135273‐74‐4
Tgm2: transglutaminase 2
TMT: tandem mass tag
WB: western blot

## 1 Introduction

Aging remains a serious risk factor in the development of neurodegenerative diseases, necessitating profound exploration into the mechanisms of neural aging. Senescent cells, recognized as one of the hallmarks of aging (López‐Otín et al. 2023), play a pivotal role in the etiology of neurodegenerative conditions like Alzheimer's disease (AD). Microglia, resident immune cells in the central nervous system (CNS), account for about 12% of the total brain cells (Van Hove et al. 2019). Aged‐like microglia disrupt brain homeostasis and phagocytic function, ultimately accelerating neuronal damage. The senescence‐associated secretory phenotype (SASP) of senescent microglia can accelerate the aging of neighboring cells by altering the cellular microenvironment and collaborating with the immune system (Li, Li, Jin, et al. 2023). Increasing dependence on healthy microglia for myelin integrity is observed with aging due to the worsening myelin pathology caused by microglia depletion. The decline in microglia function with age may lead to demyelination, impair learning and memory abilities, and accelerate the progression of neurodegenerative diseases (McNamara et al. 2023). Consequently, microglia have been identified as a potential therapeutic target for neurodegenerative diseases (Gao et al. 2023).

Transglutaminase 2 (Tgm2) is a calcium (Ca^2+^)‐dependent bifunctional enzyme with both transglutaminase activity and GTPase activities (Al‐U'datt et al. 2022). The expression and activity of Tgm2 are regulated by NF‐κB and IL6 (Al‐U'datt et al. 2022; Tatsukawa and Hitomi 2021). Microglia are the main source of Tgm2 in the brain, and the previous study has found that knockout of Tgm2 in microglia in mice leads to synaptic remodeling impairment and cognitive decline, indicating that Tgm2 is crucial for normal neural development (Liu et al. 2023). However, the specific role and underlying mechanisms of Tgm2 in senescent microglia remain unclear.

During the aging process, the activity of NF‐κB increases, leading to chronic inflammation (Tatsukawa and Hitomi 2021). Aberrant activation of the NF‐κB pathway is associated with the development of age‐related diseases such as obesity (Grun et al. 2023) and neurodegenerative diseases (Wei et al. 2023). The activation of NF‐κB upregulates the expression of Tgm2 and promotes SASP factors such as IL1α and IL6 (Al‐U'datt et al. 2022; Li, Sun, et al., 2023; Tatsukawa and Hitomi 2021). The expression of Tgm2 is also regulated by inflammatory factors (Jia et al. 2020; Oh et al. 2011; Yen et al. 2010; Zhang and McCarty 2017).

In this study, we found that Tgm2 is highly expressed in senescent microglia. Moreover, we demonstrated that Tgm2 inhibitors reduced SASP in vitro and alleviated age‐associated phenotypes in aged mice. Therefore, Tgm2 inhibitors hold promise as novel prophylactics or senomorphics for preventing aging‐related pathologies. Overall, our study shed light on the function and action mechanisms of Tgm2 in microglial senescence and offered new insights for the diagnosis and treatment of age‐related neurodegenerative diseases.

## 2 Materials and Methods

### 2.1 Cell Culture

BV2 cells, 293F cells, and 293 T cells (sourced from the cell bank of the Chinese Academy of Sciences) were cultured in DMEM (Wisent, Montreal, Canada). The culture medium was supplemented with 10% fetal bovine serum (FBS) (PAN‐Biotech, Germany) and 1% penicillin/streptomycin (Wisent, Montreal, Canada). Cells were cultured in a humidified incubator maintained at 37°C with 5% CO_2_.

Cys‐D (Cat# A53299, China) was purchased from OKA, Tg2‐IN1 (Cat# HY117678, USA) was purchased from MCE, and etoposide (Cat# SC0173, China) was purchased from Beyotime.

### 2.2 Animal Experiments

Mice experiments were performed in the animal facility of Tsinghua University (Beijing, China) with approval of the Institutional Animal Care and Use Committee of Tsinghua University (approval number: 22‐DHT3). C57BL/6J mice were purchased from Jackson Laboratory through the Laboratory Animal Research Center and housed in the animal facility of Tsinghua University with ad libitum access to diet and water. Animal rooms were maintained at 20°C–22°C with 30%–70% relative humidity and a 12‐h light/dark cycle.

Using a dose of 200 mg/kg, Cys‐D was administered by gastric gavage to 16‐month‐old C57 mice (*n* = 8) every other day for 2 months. A control group of mice (*n* = 8) received sterile water via gavage during the same period. Within 2 weeks of completing the Cys‐D treatment, the mice were individually subjected to a series of behavioral tests, including the rotarod test, grip strength test, open‐field test, and Y‐maze test.

### 2.3 Isolation of Primary Senescent Microglia

The Single Cell Suspension Preparation Instrument (DCS‐400, RWD Life Science Co Ltd., China) was used to prepare single‐cell suspensions from the brains of 22‐month‐old mice (*n* = 3), following the program “M‐NeoBrain Heater‐1.” The CD11b + Cell Sorting Kit (K1306‐10, RWD Life Science Co Ltd) was employed to isolate microglial cells from single‐cell suspensions. The isolated cells were stained with fluorescent antibodies: FITC anti‐mouse/human CD11b (M1/70, Biolegend) and IBA1/AIF‐1 (E4O4W, CST). Subsequently, flow cytometry was performed to sort and obtain relatively pure primary microglial cells. These primary microglial cells were further cultured for 3 weeks, and senescent microglia were identified using the SPiDER‐βGal assay (SG02, Dojindo). All cell sorting procedures were performed on the Bigfoot Spectral Cell Sorter (Thermo Fisher Life Science Co Ltd., USA).

### 2.4 Western Blotting

Cells were lysed in RIPA lysis buffer (Cat# P0013K, Beyotime, China) added with protease inhibitors cocktail. Equal volumes of protein were separated by 12% sodium dodecyl sulfate–polyacrylamide gel electrophoresis (SDS‐PAGE) and electrotransferred onto a polyvinylidene difluoride (PVDF) membrane. Primary rabbit anti‐Tgm2 (Cell Signaling Technology, Danvers, USA), rabbit anti‐P105, rabbit anti‐P65/Rel, anti‐phospho‐P65 (Ser 536), rabbit anti‐H3 (Abcam, Cambridge, UK), rabbit anti‐IκBα, rabbit anti‐phospho‐IκBα (Ser 32) (HuaBio, Hangzhou, China), rabbit anti‐P50, rabbit anti‐phospho‐P50 (Ser 337) (Proteintech, Chicago, USA), rabbit anti‐β‐actin, secondary anti‐rabbit HRP‐IgG antibodies (Cell Signaling Technology, Danvers, USA), and M5 HiClear Prestained Protein Ladder (Mei5bio, Beijing, China) were used for immunoblotting.

BV2 cells were subjected to nuclear and cytosolic fractionation using the Nuclear/Cytosol Fractionation Kit (invent, SC003), following the protocol recommended by the manufacturer.

### 2.5 Tandem Mass Tag (TMT)‐Based Quantitative Proteomic Analysis

#### 2.5.1 Sample Preparation

The control BV2 cells and the senescent BV2 cells were lysed by RIPA lysis buffer (Cat# P0013K, Beyotime, China) added with protease inhibitors cocktail, and the supernatant was added to acetone in a 1:5 volume at −30°C for precipitation and dissolved with 8 M urea. Take 150 μg of protein per sample and digest with trypsin at 37°C overnight for 16 h. Tryptic peptides were desalted and labeled with the TMT 6‐plex reagents (Thermo Scientific, Cat# 90110). Then, the mixed labeled peptides were subjected to LC–MS/MS analysis.

#### 2.5.2 LC–MS/MS Analysis

LC–MS/MS analysis was performed as described previously (Wang et al. 2024; Yang et al. 2021). Labeled peptides were injected onto a UHPLC 3000 system coupled to a Thermo Scientific Orbitrap Exploris 480 mass spectrometer using a C‐18 analytical column (300 Å, 5 μm, Thermo Fisher Scientific, USA). The peptides were eluted with a gradient elution program at a flow rate of 0.300 μL/min. Mobile phase A consisted of 0.1% formic acid, and mobile phase B consisted of 100% acetonitrile and 0.1% formic acid. The mass spectrometer was operated in the data‐dependent acquisition (DDA) mode using Xcalibur 4.5.445.18 software. MS1 spectra were acquired at a mass range of 300–1800 *m/z* with a resolution of 60,000. The spray voltage was set at 2100 V, and the automatic gain control (AGC) target was set to 3e^6^. For MS2 scans, the top 40 most intense precursor ions were fragmented in the HCD collision cell at a normalized collision energy of 32% using a 0.4 Da isolation window. The dynamic exclusion duration was set to 15 s, and the AGC target was set to 1e^5^ while the maximum injection time was set to 100 ms.

#### 2.5.3 Protein Identification and Quantification

The raw mass spectrometry data were searched against the *Homo sapiens* database by the Proteome Discoverer 2.3 software. Raw data of LC–MS/MS have been uploaded to the iProx website (https://www.iprox.org). The accessible number is IPX0008322001.

### 2.6 Cross‐Linking Identification by MS

A PVDF membrane of separated proteins was excised for in‐gel digestion, and proteins were identified by mass spectrometry. Briefly, proteins were reduced with 25 mM dithiothreitol (DTT) and alkylated with 55 mM iodoacetamide. In‐gel digestion was performed using sequencing grade‐modified trypsin in 50 mM ammonium bicarbonate at 37°C overnight. The peptides were extracted twice with 1% trifluoroacetic acid in 50% acetonitrile aqueous solution for 30 min. The peptide extracts were then centrifuged in a SpeedVac to reduce the volume.

The mass spectrometer was run under DDA mode. The survey of full‐scan MS spectra (200–1500 *m*/*z*) was acquired in the Orbitrap with 60,000 resolutions. The AGC target of 3e^6^ and the maximum injection time of 25 ms. Then, the precursor ions were selected into collision cell for fragmentation by higher‐energy collision dissociation, the normalized collection energy was 30. The MS/MS resolution was set at 15,000, the AGC target of 5e^4^, the maximum injection time of 22 ms, and dynamic exclusion was 30 s.

Raw data were searched using the pLink2 software against IκBα sequences with trypsin specificity. The search parameters were as follows: peptides mass tolerance of 20 ppm; MS/MS tolerance of 0.02 Da; Peptide length: 4–100 aa; three missed cleavages allowed; oxidation on Met as the dynamic modification; and carbamidomethylation on Cys as static modification. A crosslinker monoisotopic mass of −17.026 Da (Q‐K = −17.026 Da) was manually added.

### 2.7 Cloning, Expression, and Characterization of Tgm2 Protein

Tgm2 plasmid was purchased from Wuhan Miaoling Biotechnology Co. Ltd. Oligonucleotide primers:

(5′‐GCTAGCGTTTAAACGGGCCCGCCACCATGGCAGAGGAGCTG‐3′) and (5′‐GCGGTTTAAACTTAAGCTTCTAATGATGATGATGATGATGGGCCGGGCCGATGATAAC‐3′) were designed for PCR amplification of Tgm2 fragments. Primer pairs (5′‐AAGCTTAAGTTTAAACCGC‐3′) and (5′‐GGGCCCGTTTAAACGCTAGC‐3′) were used to amplify the pCDNA3.1 vector fragment. The two fragments were ligated by using Seamless Cloning Kit (Beyotime, D7010s). After sequence validation, the resulting plasmid constructs were used to produce the recombined Tgm2 proteins in HEK‐293F cells. Before transfection, the fresh culture medium was replaced, and the final concentration of the expression vector plasmid DNA in the transfection volume was 3 μg/mL, with a final concentration of PEI at 9 μg/mL. After 24 h of transfection, fresh culture medium was added at a 1:1 ratio. After 3 days of culture, cells were collected by centrifugation at 4000 rpm for 15 min. The media were discarded and cells were resuspended in PBS buffer. Initially, cells were disrupted using a low‐temperature ultrahigh‐pressure cell disruptor at 800 MPa for 30 min and centrifuge at 17,000 rpm for 1 h. Subsequently, the target protein was purified using a Ni‐NTA column on an AKTA purification platform. After concentration and buffer exchange on Centricon‐10 (Millipore), the proteins in PBS buffer were aliquoted, flash frozen in liquid nitrogen, and then stored at −80°C. The purified proteins were separated by 12% SDS‐PAGE under reducing conditions and stained with Coomassie brilliant blue. Finally, the target protein was identified using WB (6 × His antisera as the primary antibody and rabbit antisera as secondary antibody) quantified using BCA (Beyotime).

### 2.8 Immunofluorescence

BV2 cells cultured on glass coverslips were fixed in 4% paraformaldehyde for 20 min. Cells permeabilized in PBS with 0.5% Triton X‐100 for 15 min at room temperature. Blocked as above, and then incubated with anti‐Tgm2 antibody and anti‐p65 antibody at 4°C overnight. Then, it was incubated with a secondary antibody of Alexa Fluor 488‐conjugated anti‐rabbit IgG secondary antibody (1:200) (Abcam, ab150077) or Alexa Fluor 647‐conjugated anti‐mouse IgG secondary antibody (1:200) (Abcam, ab150115). Finally, the nuclei were stained with DAPI. Images were captured with LSM980 (ZEISS, Germany).

### 2.9 Cell Viability Assay

Cells were seeded and cultured in 96‐well plates. After treatment, the cell survival rate was measured using CCK8 reagent (Cat# K1018, ApexBio Technology, USA) according to the manufacturer's instructions. Briefly, CCK8 reagent was added into the wells, then the plates were incubated in a cell incubator for 1.5 h. The absorbance at 450 nm was measured to calculate cell viability.

### 2.10 Real‐Time Quantitative PCR Analysis

Total RNA was isolated from cells by using Trizol reagent (TIANGEN, Beijing, China). Liver tissues were added into Trizol reagent and homogenized with a grinding rod. Then, cDNA was synthesized using a commercially available kit following the manufacturer's instructions (CWBIO, Beijing, China). qPCR analysis was performed using SYBR green reaction mixture (CWBIO, Beijing, China) and Roche LightCycler 96 System (Roche, Basel, Switzerland). β‐Actin was used as the internal control for relative quantification. Primers used in qPCR are listed in Data S1.

### 2.11 Hematoxylin–Eosin Staining

Tissues were fixed with 4% paraformaldehyde overnight. Then, tissues were embedded in paraffin. Five‐micrometer sections were prepared, deparaffinized, and hydrated sequentially, stained with HE. The sections were dehydrated by gradient alcohol, transparentized with xylene, and mounted with neutral resin for light microscopy.

### 2.12 Immunohistochemistry Staining

Tissues were fixed with 4% paraformaldehyde overnight. Then, tissues were embedded in paraffin according to the standard protocol. Five‐micrometer sections were prepared, deparaffinized, and hydrated sequentially. Antigen retrieval was performed using EDTA buffer (Servicebio), and endogenous peroxidase was blocked. The slides were blocked with 3% BSA at room temperature for 30 min, before incubation with Tgm2 primary antibody at 4°C overnight. After being washed with PBS twice, slides were incubated with secondary antibody (Servicebio). The signal was detected by DAB staining. Nuclear was stained by hematoxylin. The sections were dehydrated by gradient alcohol, transparentized with xylene, and mounted with neutral resin for light microscopy.

### 2.13 SA‐β‐Galactosidase Staining

Cellular senescence was assessed by examining the activity of β‐galactosidase using SA‐β‐Gal staining kit (Cat# C0602, Beyotime, China) according to the manufacturer's instructions. Briefly, the cells in 12‐well plates were fixed with fixation solution for 10 min, then washed with PBS twice and stained with SA‐β‐Gal solution at 37°C overnight. Then, the samples were imaged by light microscopy (Nikon‐90i, Japan), and the blue‐stained cells were identified as senescent cells.

### 2.14 Rotarod Test

Mice were trained on the Rotarod (Ugo Basile, Italy) at a speed of 10 rpm for 3 days. During the test, the speed of the Rotarod was 10 rpm, and the time on the rod before falling was recorded.

### 2.15 Grip Strength Test

The maximal grip strength was measured using a grip strength meter (Ugo Basile, Italy). The animals were put on a metal grid, and their tail was caught and pulled back. The force exerted on the grid by the animals' legs was measured in grams. Three tests were performed, and the mean of the three measurements was calculated.

### 2.16 Y‐Maze Test

The Y‐maze test serves as a tool to evaluate the short‐term spatial working memory capabilities of rodents. The Y‐maze test apparatus consists of three arms that intersect at 120° angles, forming a shape reminiscent of the letter Y. Each arm has walls that are 11 cm high, 10 cm wide, and 31 cm long. Herein, the Y‐maze test was conducted according to a previously reported protocol (Choi et al. 2024). The cumulative duration in the food arm and frequency of leaving the food arm was recorded for 5 min. These data are statistically analyzed by automated behavioral video software (EthoVision XT 11.5, Noldus Information Technology b.v., Netherlands).

### 2.17 Open Field Test

The open field was utilized to assess the effects of Cys‐D treatment on aged mice. The box (60 × 60 × 60 cm) was placed directly under the camera, and the mice were allowed to freely move within the box for 5 min. After the test of every mouse, the apparatus was cleaned and sterilized, and the experimental chamber was wiped with 75% alcohol. The movement of the mice was recorded on video. The distance walked and the average speed were analyzed using the EthoVision XT 11.5.

### 2.18 Construction of Tgm2 Knockdown BV2 Cell Line

Tgm2 shDNA of the mouse was subcloned into lentivirus vector TRC2‐pLKD‐puro to construct a lentiviral vector TRC2‐pLKD‐Tgm2‐puro. Then, lentiviral vectors were packaged and the titer was determined. BV2 cells were transfected with the constructed TRC2‐pLKD‐Tgm2‐puro (Tgm2‐KD or TKD group) and TRC2‐pLKD‐puro (NC group) as controls. Tgm2‐KD and NC groups were identified by RT‐qPCR and WB.

Tgm2 gene shRNA sequence:

CCGGCCTGGTCTTTATCCTAAGATACTCGAGTATCTTAGGATAAAGACCAGGTTTTT.

### 2.19 Statistical Analysis

Student's *t*‐test was used to compare the two groups. One‐way ANOVA was used for multiple comparisons. *p* < 0.05 was considered statistically significant. Data were presented as the means ± SD. GraphPad Prism software (version 9.0) was used for statistical analysis.

## 3 Results

### 3.1 Tgm2 is Highly Expressed in Senescent Microglia Both In Vivo and In Vitro

**FIGURE 1:**
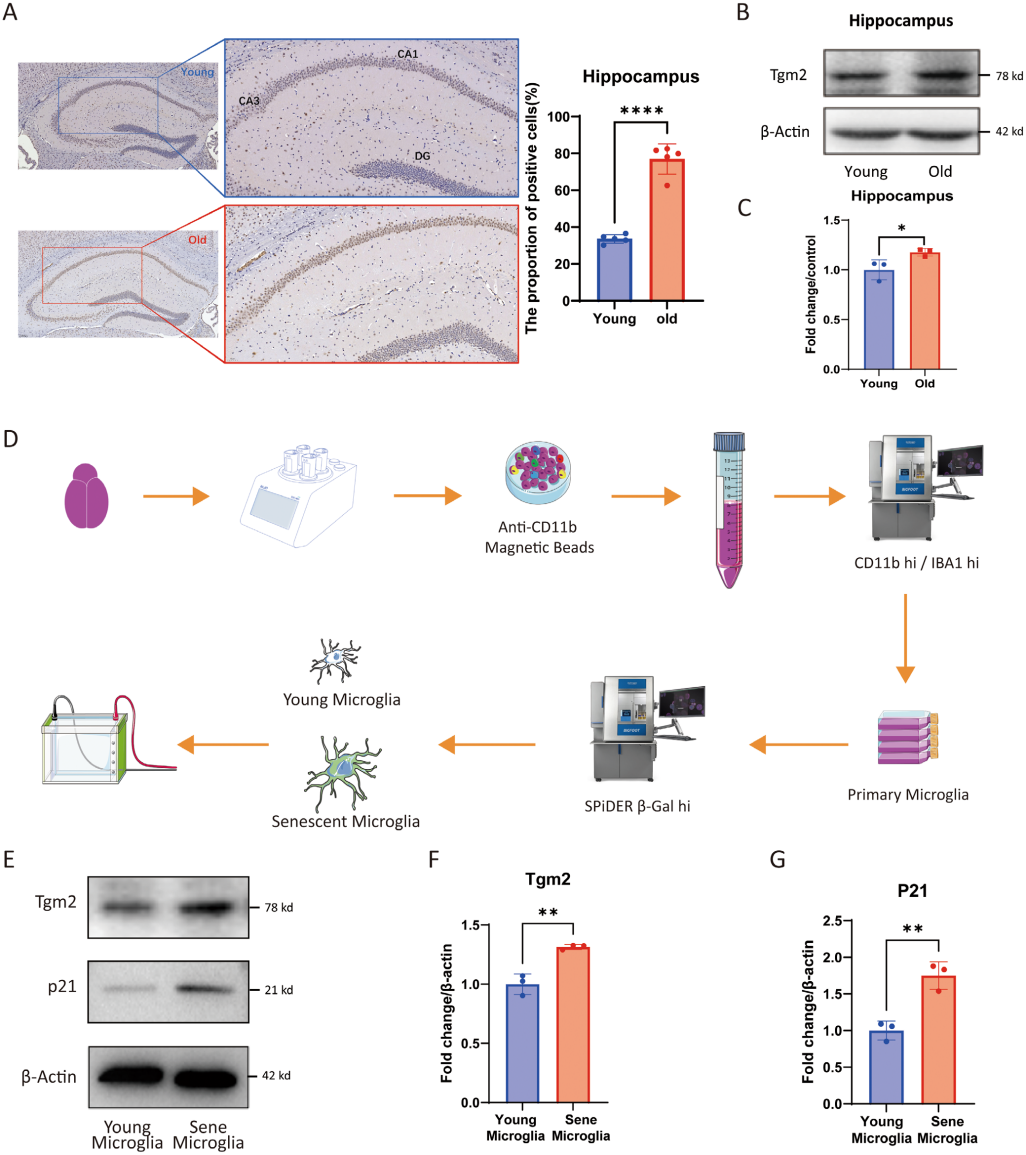
Tgm2 is highly expressed in senescent microglia. (A) Representative immunohistochemical images of Tgm2 protein in the hippocampal tissue of young (4‐month) and old (22‐month) C57/B6 mice. (B, C) The protein abundance relative expression level of Tgm2 in the hippocampus of C57/B6 mice by western blotting analysis (*n* = 3). (D) A workflow for isolating and identifying senescent primary microglia from 22‐month‐old mice (*n* = 3). (E) Western blot analysis of Tgm2 and P21 protein levels in primary microglia. (F, G) The protein abundance relative expression level of Tgm2 and P21 by western blot analysis (*n* = 3). Young, Young mice or young cells; Sene, Senescent cells. Data were analyzed by Student's *t*‐test. *p* > 0.05 is indicated by ns; *p* < 0.05 is indicated by ⁎; *p* < 0.01 is indicated by ⁎⁎; *p* < 0.0001 are indicated by ⁎⁎⁎⁎.

As previously reported, microglia are the primary source of Tgm2 in the brain (Giera et al. 2018; Liu et al. 2023). To explore the role and mechanism of Tgm2 in the brain aging process, we performed IHC and WB experiments on mouse brain tissue. Immunohistochemical staining demonstrated increased expression of Tgm2 in the CA1 and CA3 regions of the hippocampus in aged C57/B6 mice (Figure 1A). Furthermore, WB analysis confirmed that Tgm2 was highly expressed in the hippocampus of aged mice (*p* < 0.05, Figure 1B,C). These results suggested that Tgm2 was upregulated in aged mice. Additionally, we utilized flow cytometry in combination with cell sorting (Figure 1D and Data S1) to determine that Tgm2 was highly expressed in primary senescent microglia (*p* < 0.01, Figure 1E–G).

**FIGURE 2:**
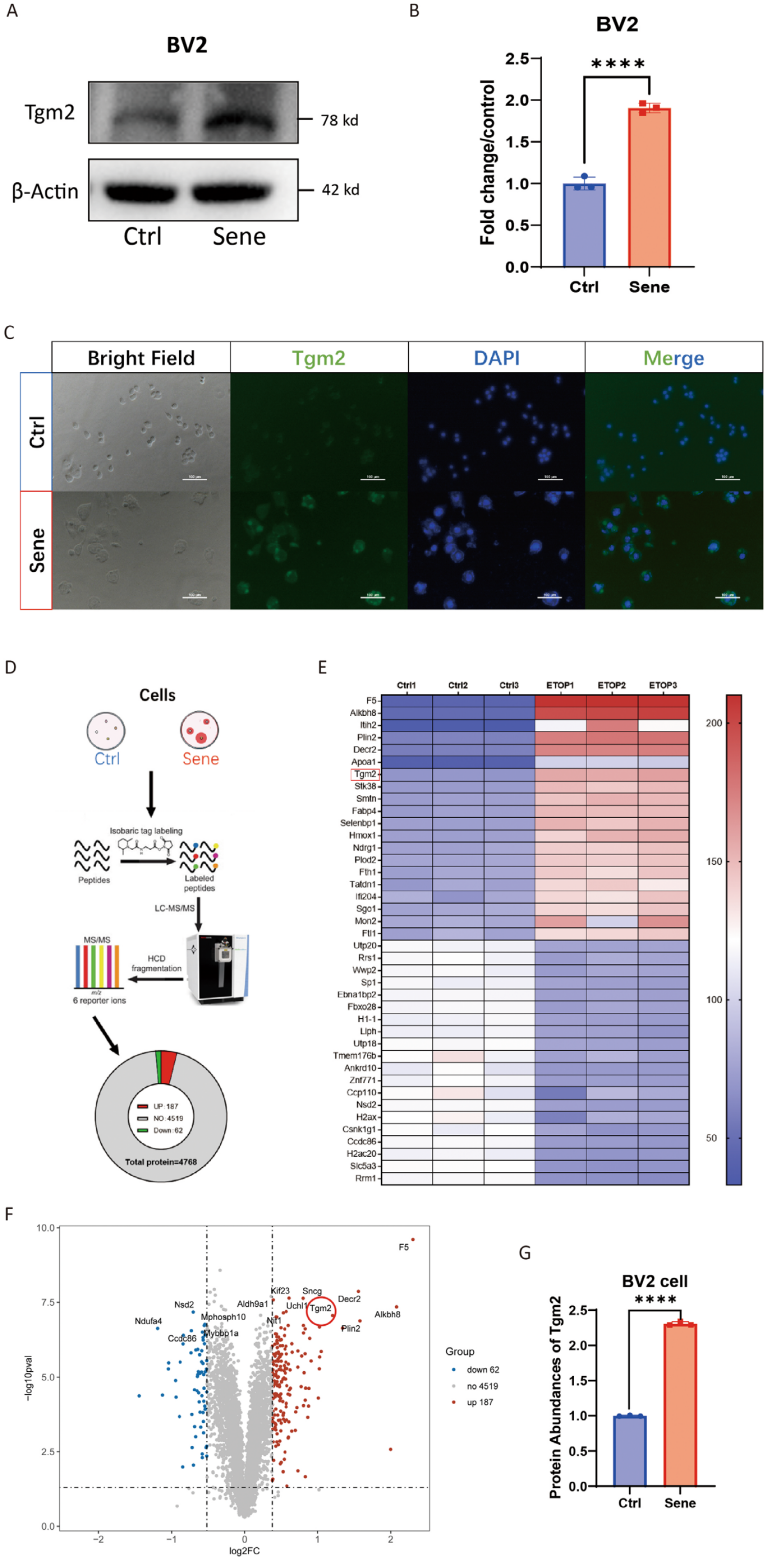
Tgm2 is highly expressed in senescent BV2 cells. (A) Western blotting analysis of Tgm2 in senescent BV2 cells. (B) Graphs represent the quantification of the Tgm2 blots (*n* = 3). (C) Immunofluorescence images of Tgm2 protein in control and senescent BV2 cells. Scale bar: 100 μm. (D) The TMT quantitative proteomics analysis workflow for senescent BV2 cells. (E, F) The heatmap and volcano plot depicted the difference in protein levels between control cells (Ctrl) and senescent cells (Sene). (G) The relative protein abundance of Tgm2 in BV2 cells was determined by TMT quantitative proteomics analysis (*n* = 3). Ctrl, control cells; Sene, senescent cells. Student's *t*‐test analyzed data. *p* < 0.0001 are indicated by ⁎⁎⁎⁎.

In addition, we established an in vitro model of senescent microglia by treating the BV2 cell line with etoposide. WB (*p* < 0.0001, Figure 2A,B) was used to confirm the high expression of Tgm2 in senescent BV2 cells. IF results also confirmed the high expression of Tgm2 in senescent BV2 cells, primarily localized in the cytoplasm (Figure 2C). Moreover, we performed a six‐plex TMT quantitative proteomics analysis on senescent BV2 cells (Figure 2D), the heat map (fold change > 2.2, Figure 2E), and volcano map (Figure 2F) revealed that Tgm2 ranked seventh among the 187 upregulated proteins (*p* < 0.001, Figure 2G). These results suggest that Tgm2 is highly expressed in senescent microglia both in vivo and in vitro.

### 3.2 The NF‐κB Pathway is Activated in Senescent Microglia and the Brain Tissues of Aged Mice

**FIGURE 3:**
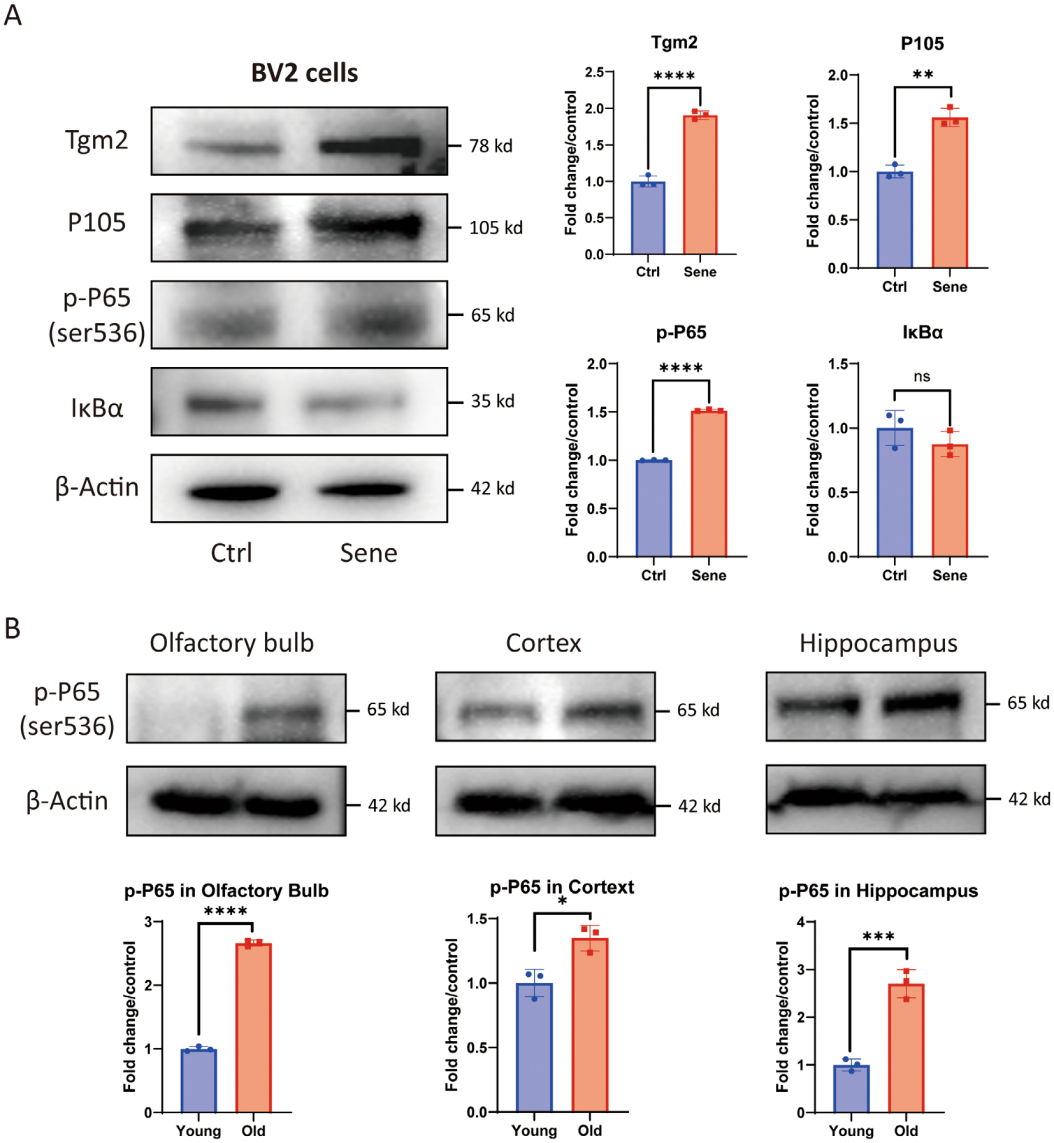
The NF‐κB pathway is activated in senescent BV2 cells and the brain tissues of aged mice. (A) Western blotting analysis of Tgm2, P105, p‐P65, and IκBα protein levels in senescent BV2 cells (*n* = 3). (B) Western blotting analysis of Tgm2, P105, p‐P65, and IκBα protein levels in the olfactory bulb, cortex, and hippocampus tissues of C57/B6 mice (*n* = 3). Data were analyzed by Student's *t*‐test. *p* > 0.05 is indicated by ns; *p* < 0.05 is indicated by ⁎; *p* < 0.01 is indicated by ⁎⁎; *p* < 0.001 is indicated by ⁎⁎⁎; *p* < 0.0001 is indicated by ⁎⁎⁎⁎.

The NF‐κB pathway was enriched in the results of TMT quantitative proteomics for senescent microglia by KEGG pathway analysis (Data S1), and the NF‐κB pathway plays a critical role in the brain aging process (Lee et al. 2004; Liu and Mouradian 2024). The WB results of senescent cells revealed an increase in the expression of Tgm2, as well as an upregulation of P105 and p‐P65(Ser 536) in the NF‐κB pathway. However, the expression of IκBα was decreased (Figure 3A). In addition, it was found that compared to 3‐month‐old C57/B6 mice, the protein content of p‐P65 (Ser 536) in the olfactory bulb, cortex, and hippocampal tissues of 20‐month‐old mice also significantly increased (Figure 3B). These results indicated that the NF‐κB pathway was activated in senescent microglia as well as in the brain tissue of aged mice.

### 3.3 Tgm2 Inhibitors Can Reduce the Nuclear Translocation of NF‐κB in Senescent Microglia

**FIGURE 4:**
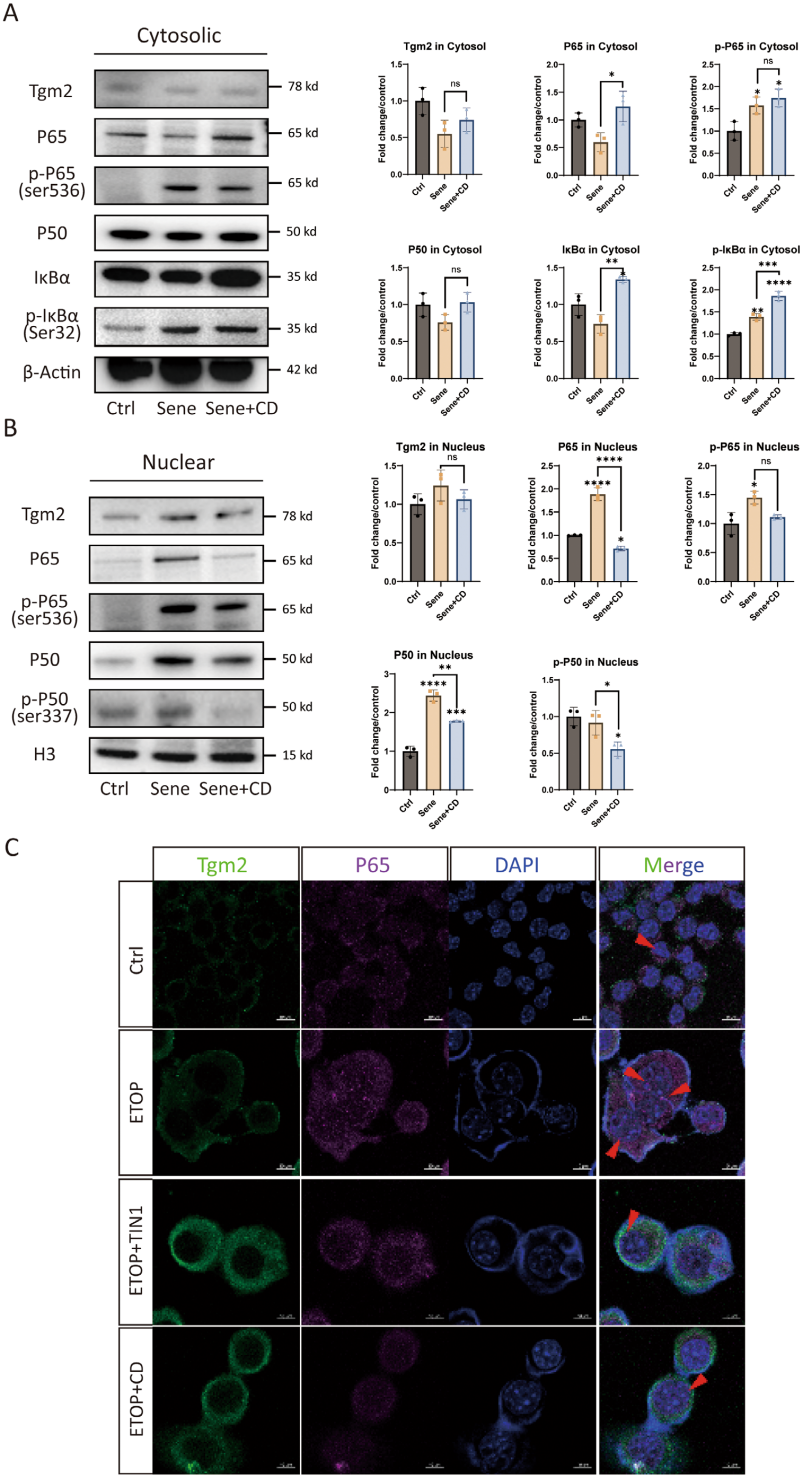
Tgm2 inhibitors (CD and TIN1) treatment reduces the NF‐κB nuclear translocation in senescent BV2 cells. (A) Western blotting analysis of Tgm2, P65, p‐P65, P50, p‐IκBα, and IκBα protein levels in cytosol of BV2 cells (*n* = 3). (B) Western blotting analysis of Tgm2, P65, p‐P65, P50, p‐P50, and IκBα protein levels in nucleus of BV2 cells (*n* = 3). (C) Tgm2 inhibitor treatment reduces the P65 in the nucleus of senescent BV2 cells by immunofluorescence. TIN1: TG‐2‐IN‐1 (CAS No.: 135273‐74‐4) is a Tgm2 inhibitor. ETOP + TIN1: BV2 cells were treated with 10 μM etoposide and 200 μM TIN1 together. Scale bar: 10 μm. Data were analyzed by one‐way ANOVA. *p* > 0.05 is indicated by ns; *p* < 0.05 is indicated by ⁎; *p* < 0.01 is indicated by ⁎⁎; *p* < 0.001 is indicated by ⁎⁎⁎; *p* < 0.0001 is indicated by ⁎⁎⁎⁎.

To investigate the role of Tgm2 in senescent BV2 cells, we used a Tgm2 inhibitor Cys‐D to treat senescent cells. We extracted cytosolic and nuclear proteins from normal BV2 cells (labeled as Ctrl), senescent cells (labeled as Sene), and Cys‐D‐treated senescent cells (labeled as Sene+CD) for WB analysis. The results showed that the protein levels of p‐P65 (Ser 536) and p‐IκBα (Ser 32) were increased in the cytosol of senescent BV2 cells compared to the Ctrl group. The cytosolic protein of P65 and IκBα were increased in the Sene+CD group compared to the Sene group (Figure 4A). Additionally, it was observed that the protein levels of P65, p‐P65 (Ser 536), and P50 were elevated in the nuclei of senescent BV2 cells from the Sene group. However, in the nuclei of the Sene+CD group, the protein levels of P65, P50, and p‐P50 (Ser 337) were reduced, while the protein levels of Tgm2 and p‐P65 (Ser 536) remained unchanged (Figure 4B).

Airyscan IF microscopy was used to confirm further the enhanced nuclear translocation of P65 in senescent microglia, which was attenuated by the administration of the Tgm2 inhibitors TIN1 and CD (Figure 4C). In conclusion, it was observed that the nuclear translocation of NF‐κB increased in senescent microglial cells, and the Tgm2 inhibitor reduced the nuclear translocation of NF‐κB.

### 3.4 Tgm2 Inhibition or Knockdown Reduces SASP of Senescent Microglia

**FIGURE 5:**
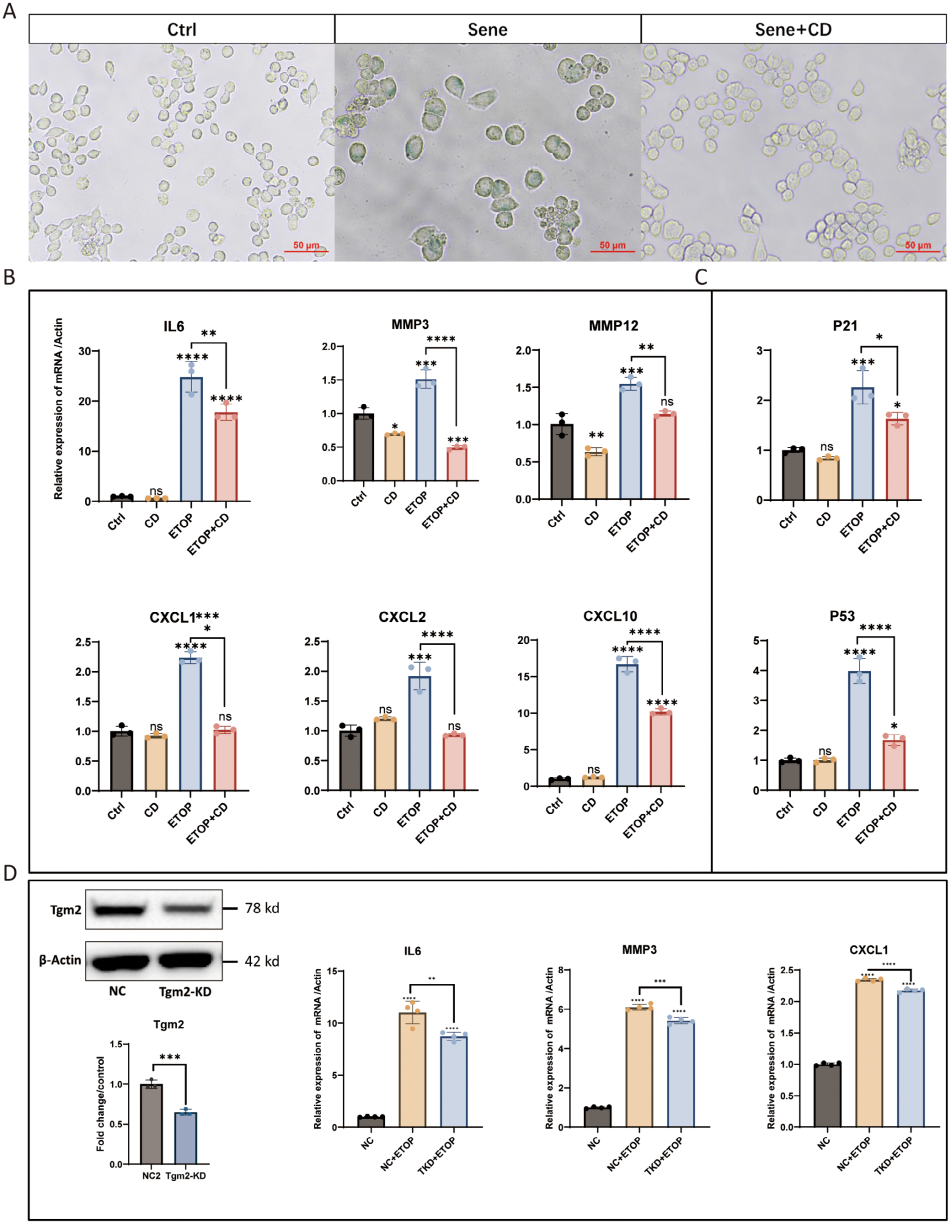
CD treatment reduces SASP factors in senescent BV2 cells. (A) SA‐β‐Gal staining results of BV2 cells. Ctrl, control cells; Sene, Senescent cells. Sene+CD: BV2 cells were treated with 10 μM etoposide and 500 μM CD together. Scale bar: 50 μm. (B, C) RT‐qPCR was used to measure mRNA levels of *IL6*, *MMP3*, *MMP12*, *CXCL1*, *CXCL2*, *CXCL10*, *P21*, and *P53* (*n* = 3). Ctrl, control cells. CD: BV2 cells were treated with 500 μM CD. ETOP: BV2 cells were treated with 10 μM etoposide to induce DNA damage‐induced senescence. ETOP+CD: BV2 cells were treated with 10 μM etoposide and 500 μM CD together. (D) RT‐qPCR was used to measure mRNA levels of *IL6*, *MMP3*, and *CXCL1* (*n* = 4). NC, negative control BV2 cells. NC + ETOP: Negative control BV2 cells were treated with 10 μM etoposide. TKD + ETOP: BV2 cells with Tgm2 knockdown were treated with 10 μM etoposide. Data were analyzed by one‐way ANOVA or Student's *t*‐test. *p* > 0.05 is indicated by ns; *p* < 0.05 is indicated by ⁎; *p* < 0.01 is indicated by ⁎⁎; *p* < 0.001 is indicated by ⁎⁎⁎; *p* < 0.0001 is indicated by ⁎⁎⁎⁎.

To further explore the role of Tgm2 in senescent microglia, we used Tgm2 inhibitor and knocked down Tgm2 expression. The results showed that senescent BV2 cells exhibited enlarged morphology and positive staining for SA‐β‐gal, whereas the Cys‐D could reduce senescent BV2 cells (Figure 5A). The increase in SASP levels is a crucial hallmark of senescent cells (Birch and Gil 2020; Tchkonia et al. 2013). Therefore, we analyzed the expression of SASP factors using RT‐qPCR. The results revealed that SASP was upregulated in senescent BV2 cells, and Cys‐D decreased the relative mRNA expression levels of SASP factors, including interleukin‐6 (*IL6*), matrix metallopeptidase 3 (*MMP3*), matrix metallopeptidase 12 (*MMP12*), and chemokines such as *CXCL1*, *CXCL2*, and *CXCL10* (Figure 4B). Additionally, Cys‐D also reduced the relative mRNA expression levels of *P21* and *P53* in senescent BV2 cells (Figure 4C).

To further confirm the regulatory role of Tgm2 in SASP expression in senescent BV2 cells, we constructed a Tgm2 knockdown cell line. Consistent with the results obtained using Tgm2 inhibitors, we found that Tgm2 knockdown in senescent BV2 cells significantly reduced the expression of SASP factors, such as *IL6*, *MMP3*, and *CXCL1* (Figure 4D). These results indicate that Tgm2 plays a regulatory role in SASP in senescent BV2 cells. Furthermore, Tgm2 inhibitors represent potential prophylactics or senomorphics to mitigate the SASP in senescent microglia.

### 3.5 Tgm2‐Catalyzed Cross‐Linking of IκBα Promotes NF‐κB Nuclear Translocation

**FIGURE 6:**
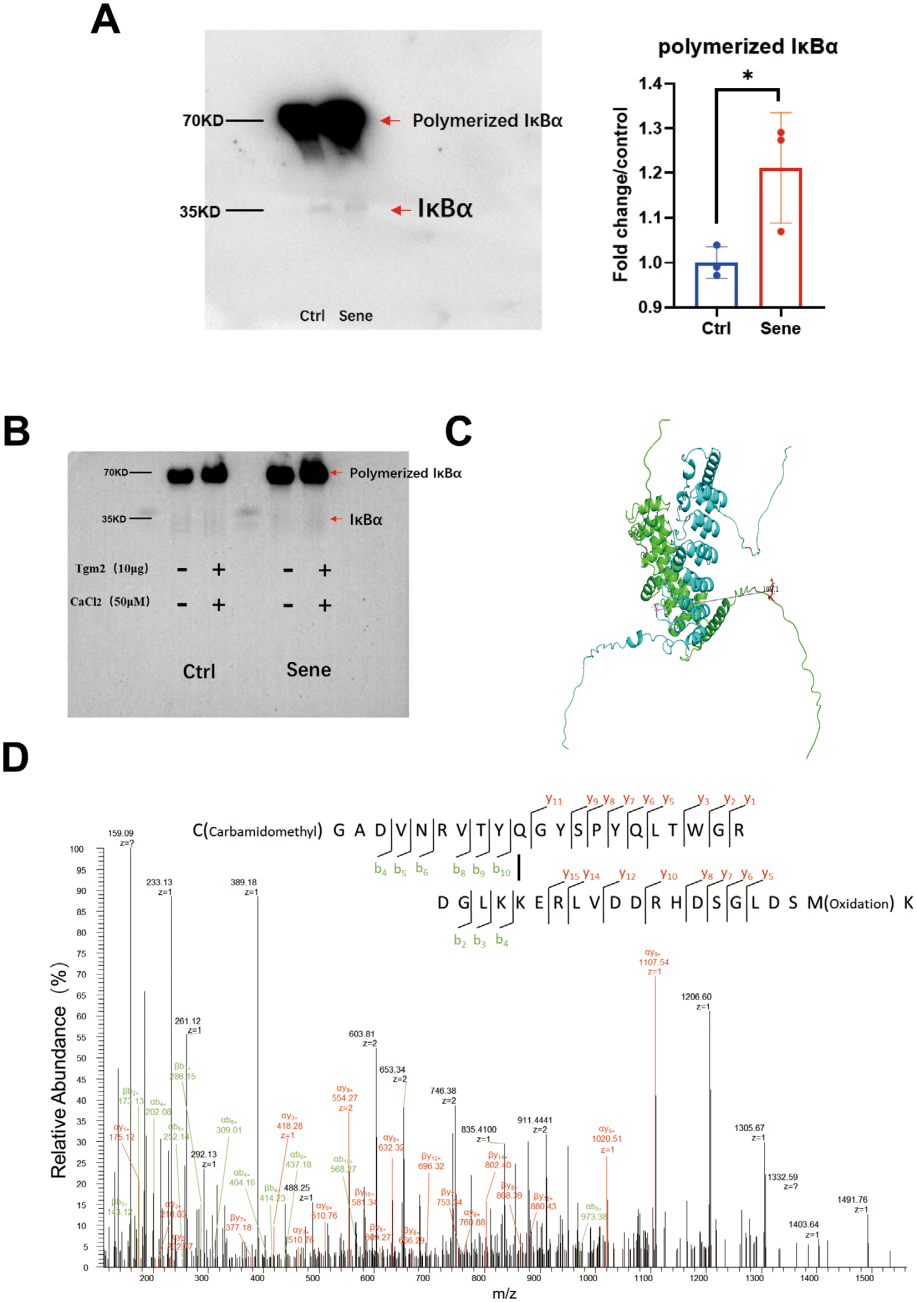
Tgm2 catalyzes the covalent cross‐linking of the residues K22 and Q248 of IκBa in the cytoplasm of BV2 cells, resulting in the dimerization of IκBa. (A) The nondenaturing western blot result for IκBα protein in the cytosol of senescent BV2 cells. (B) The nondenaturing western blot result demonstrates that Tgm2 protein promotes the cross‐linking of IκBα into dimers in the cytosol of senescent BV2 cells in vitro. (C) The IκBa dimer structure, predicted by AlphaFold 3.0, shows the two molecules are centrosymmetric. (D) The MS2 spectra of the Tgm2–cross‐linked IκBα peptide segments in the cytosol of senescent BV2 cells. Data were analyzed by Student's *t*‐test. *p* < 0.05 is indicated by ⁎.

Next, we aim to understand the mechanism underlying the regulation of SASP factors by Tgm2 in senescent BV2 cells. Tgm2 inhibitors effectively reduced the SASP level in senescent microglia (Figure 4B). IκBα protein, as an inhibitory factor for NF‐κB, was decreased in both total protein extracts (Figure 3A) and cytoplasmic protein fractions (Figure 4A) of senescent BV2 cells. IκBα is a key protein that enables Tgm2 to promote the nuclear translocation of NF‐κB. We enriched the IκBα protein in the cytoplasm of senescent BV2 cells using IκBα monoclonal antibody and then performed nondenaturing WB. We found that the content of IκB*α* dimers was significantly higher in senescent BV2 cells compared to control cells (Figure 6A). To further verify Tgm2 cross‐linking IκBα, we coincubated 10 μg purified Tgm2 protein (Data S1) and 50 μM CaCl_2_ with enriched natural IκB*α* protein in the cytoplasm of senescent BV2 cells at 37°C for 1 h. The results showed that Tgm2 promoted the cross‐linking of IκBα into dimers in the cytoplasm of senescent BV2 cells, reducing the non–cross‐linked IκBα content (Figure 6B). The structure of IκBa dimer was predicted using AlphaFold 3.0 (https://alphafold.com), revealing that the two IκBa molecules are centrosymmetric (Figure 6C). Finally, mass spectrometry identified new covalent cross‐linking sites K22 and Q248 of IκBα mediated by Tgm2 (Figure 6D). We suggest that the K22 and Q248 residues of each IκBa monomer form covalent bonds under the catalysis of Tgm2.

### 3.6 Tgm2 Inhibitor Alleviates the Aged‐Associated Phenotype in Aged Mice

**FIGURE 7:**
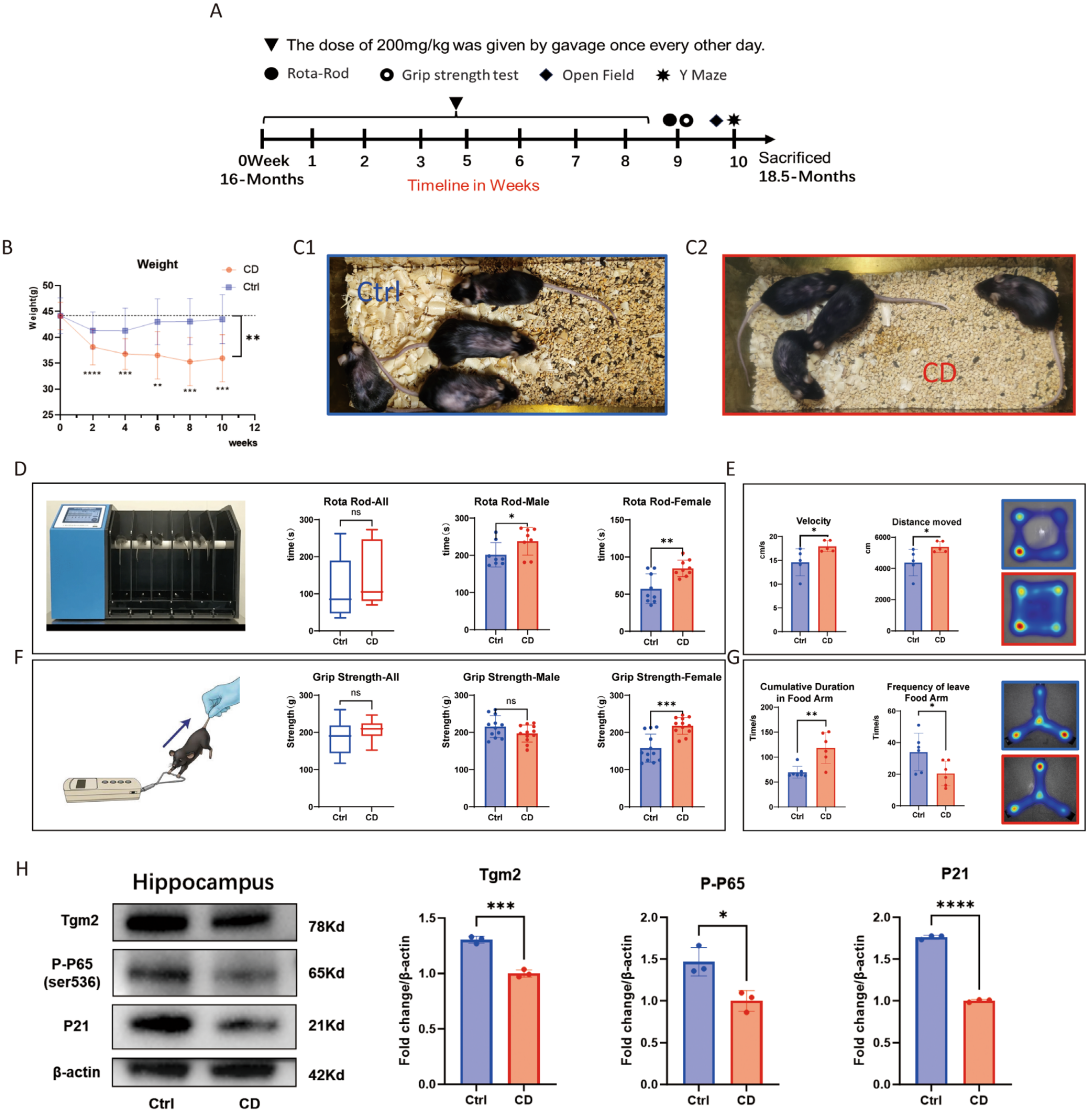
CD (Tgm2 enzyme inhibitor) can alleviate the senescent phenotype of aged mice. (A) The timeline of the experiment using CD to treat aged mice. (B) The trend of weight change in aged mice. (C) CD improves hair loss in aged mice. C1: Mouse were gavaged with H_2_O; C2: Mouse were gavaged with CD (*n* = 8). (D) The results of the rotarod test in aged mice after CD treatment. (E) The results of the open‐field test in aged mice after CD treatment. (F) The results of the grip strength test in aged mice after CD treatment. (G) The results of the Y‐maze test in aged mice after CD treatment. (H) CD reduces the levels of senescence and inflammation markers in the hippocampus. Data were analyzed by Student's *t*‐test. *p* > 0.05 is indicated by ns; *p* < 0.05 is indicated by ⁎; *p* < 0.01 is indicated by ⁎⁎; *p* < 0.001 is indicated by ⁎⁎⁎.

Based on the results showing the reduction of SASP in senescent microglia with Cys‐D, we administered a dose of 200 mg/kg Cys‐D every other day via oral gavage to 16‐month‐old mice (*n* = 8) for 2 months, while a control group of mice (*n* = 8) received sterile water via oral gavage. Within 2 weeks after the end of the gavage period, the mice underwent the rotarod test, grip strength test, open‐field test, and Y‐maze tests (Figure 7A). The Cys‐D‐treated group of mice initially showed a decrease in body weight, followed by stabilization, compared to the control group (Figure 7B). After treatment with Cys‐D, the hair of the aged mice became shiny, and the hair loss condition improved compared to that of the Ctrl group (Figure 7C). Cys‐D improved the performance of aged mice in the rotarod test, especially for female mice, whose duration on the rod increased by about 20% (Figure 7D). It did not affect the grip strength of male mice, but the grip strength of female mice increased by more than 20% (Figure 7F). Additionally, results from the open‐field test (Figure 7E) and Y‐maze test (Figure 7G) indicated that Cys‐D improved the adaptability to new environments and spatial memory of old mice. In conclusion, Cys‐D not only prevented hair loss in old mice but also partially reduced their body weight, and increased their agility without affecting their strength.

Treatment with Cys‐D significantly reduced the levels of Tgm2, p‐P65, and P21 in the hippocampal tissue of aged mice (Figure 7H). We speculate that the improvement in cognitive behavior in mice may be associated with reduced inflammation and delayed aging in hippocampal cells.

## 4 Discussion

Tgm2, as a potential biomarker for age‐related frailty (Cardoso et al. 2018), has also been proven to be associated with the enhancement of age‐related vascular matrix cross‐linking, and accelerating the aging of vascular smooth muscle cells (Wang et al. 2021), leading to vascular hardening. It serves as a marker and potential therapeutic target for vascular aging (Pinilla et al. 2021; Santhanam et al. 2010). IF staining and quantitative proteomics results also confirm the high expression of Tgm2 protein in senescent microglia from our study. In addition, a single‐cell study on immune remodeling in gallbladder cancer found that senescent Tgm2+ fibroblasts altered the tumor microenvironment through SASP, resisted immunotherapy, and promoted tumor migration (Wang et al. 2022). Tgm2 expression increased during the senescence process of cells with abundant extracellular matrix and strong migration ability, such as senescent fibroblasts and senescent macrophages. Microglia, as a type of macrophage‐like cells in the brain, exhibit significantly increased expression of Tgm2 in aged mice.

Cys‐D, a Tgm2 inhibitor, can cross the blood–brain barrier, and is commonly used in studies of brain gliomas (Zheng et al. 2023), Huntington disease (Verny et al. 2017), and Parkinson disease (Cicchetti et al. 2019). Cys‐D has been approved by the US Food and Drug Administration (FDA) for the treatment of cystinosis and pancreatic cancer, with a high safety dose range, and our data also support the safety of Cys‐D (Data S1). Treating microglia with 200 μM Cys‐D can counteract cell senescence (Figure 5A and Data S1) and significantly reduce the SASP of senescent cells (Figure 5B). The levels of SASP factors such as *IL6*, *MMP3*, and *CXCL1* were reduced in senescent BV2 cells with Tgm2 knockdown (Figure 5D). Senomorphics can suppress SASP, which drives sterile inflammation associated with aging (Lagoumtzi and Chondrogianni 2021; Li, Li, Zhang, et al. 2023). Small molecules that reduce Tgm2 activity or expression have the potential to become novel senomorphic.

Quantitative proteomics results of senescent BV2 cells found that many proteins in the NF‐κB pathway changed as expected (Data S1). NF‐κB can also bind to the Tgm2 promoter to regulate its expression (Tatsukawa and Hitomi 2021). WB experiments verified that the NF‐κB pathway is activated in senescent microglia, with increased expression of P105 and p‐P65 (Figure 3). To further confirm the activation of the NF‐κB pathway during the senescence process, we separated the cytoplasm and nucleus and detected key proteins separately. The results showed that P65 and p‐IκBα increased in the cytoplasm of senescent cells, while P50, P65, and p‐P65 increased in the nucleus (Figure 4A,B). These results also show that Cys‐D and Tgm2‐IN1 can reduce the activation of the NF‐κB pathway in senescent microglia.

Kim SY et al. simulated the cross‐linking of IκBα mediated by Tgm2 in vitro, demonstrating that IκBα is a substrate of Tgm2 and that Tgm2 can cross‐link IκBα into multimers. Furthermore, they used mass spectrometry to analyze the potential cross‐linking sites (Lee et al. 2004; Park et al. 2006). We analyzed the expression levels of proteins related to the NF‐κB pathway in the nucleus and cytoplasm of senescent BV2 cells and found that IκBα was a key protein in the Tgm2‐NF‐κB‐SASP loop. After enriching IκBα proteins in the cytoplasm using monoclonal antibodies against IκBα and performing nondenaturing WB, we discovered that the content of IκBα dimers in senescent microglia was significantly higher than in control cells (Figure 6A). Additionally, purified Tgm2 protein in vitro also promoted the cross‐linking of IκBα into dimers in the cytoplasm of senescent BV2 cells, resulting in a reduction in the content of non‐cross‐linked IκBα (Figure 6B). Some studies have reported that microglia are the primary source of Tgm2 in the brain (Liu et al. 2023; Li, Li, Jin, et al. 2023). Furthermore, single‐cell RNAseq analyses of mouse microglia also revealed high expression levels of Tgm2 in these cells (http://www.microgliatlas.com). In our study, mice administered with an oral Tgm2 inhibitor exhibited significant reductions in the protein levels of p‐P65 and P21 in the hippocampal tissue, where microglia showed high expression of Tgm2. Based on these findings, we suggest that decreasing Tgm2 content in hippocampal microglia may mitigate brain aging and reduce neuroinflammation (Figure 7).

**FIGURE 8:**
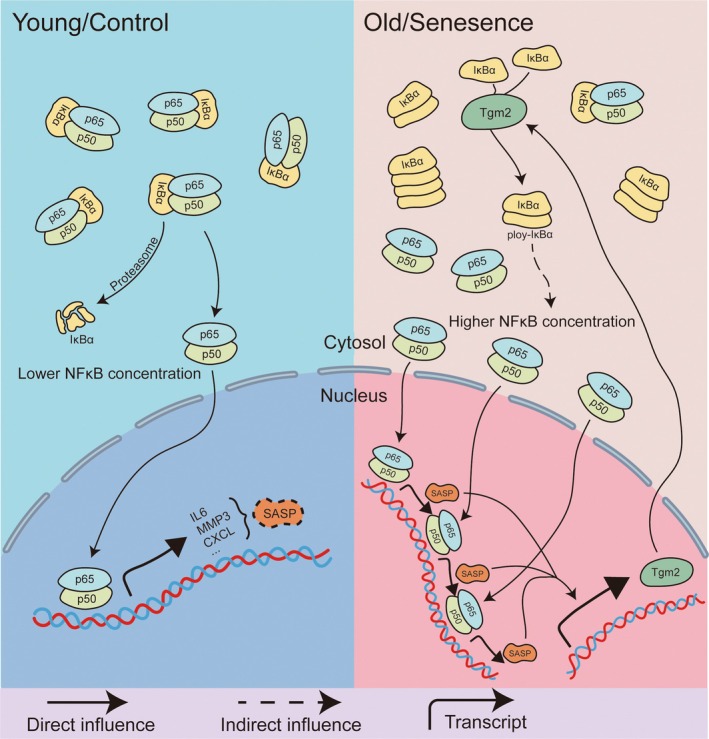
A schematic summary of the Tgm2‐NF‐κB‐SASP loop pathway in senescent microglia.

Based on these findings, the key mechanism that sustains SASP in senescent microglia is likely the cross‐linking of IκBα by Tgm2, resulting in the formation of IκBα dimers, which in turn decreases the cytoplasmic concentration of non–cross‐linked IκBα. This, in turn, facilitates the nuclear translocation of NF‐κB and promotes the expression of SASP. These inflammatory factors, such as IL6, promote the expression of Tgm2 through pathways like IL6 R/p‐STAT3 (Jia et al. 2020; Oh et al. 2011; Zhang and McCarty 2017). The expression of MMPs and Tgm2 may interfere with each other through the Wnt3a/β‐catenin/Cyclin D1 pathway (Yang et al. 2019). Taken together, we suggest a positive feedback loop among Tgm2, NF‐κB, and SASP in senescent microglia (Figure 8).

Existing senomorphics, such as metformin (Moiseeva et al. 2013) and rapamycin (Laberge et al. 2015), inhibit SASP factors at the translational level by blocking the nuclear translocation of components of the NF‐κB pathway. Drugs like roxadustat and rapamycin reduce NF‐κB nuclear translocation by interfering with the mTOR pathway, whereas Cys‐D achieves this by reducing the enzymatic activity of Tgm2 and increasing the level of non–cross‐linked IκBα. Animal experiments have also validated Cys‐D as a potential senomorphic. Administration of Cys‐D at a dose of 200 mg/kg not only improved hair and spatial memory abilities in aged mice but also reduced their body weight to a certain extent inhibiting the enzymatic activity of Tgm2 with small molecules may have the potential to reduce SASP in senescent microglia through the Tgm2‐NF‐κB‐SASP loop. This discovery may hold promise for the treatment of neurodegenerative diseases.

## Author Contributions

Z. Li, H. Deng, and W. Wei designed the research. Z. Li, T. Wang, and S. Du performed the experiments with help from Y. Zhao, Z. Miao, X. Meng, S. Yu, D. Zhang, H. Jiang, and K. Du. Y. Tang did the bioinformatics analysis. Z. Li and H. Deng wrote the manuscript. All authors reviewed, edited, and approved the manuscript.

## Conflicts of Interest

The authors declare no conflicts interest.

## Supporting information


Data S1.

## Data Availability

Data will be made available on request.
