# Supplementary material for: Tgm2‐Catalyzed Covalent Cross‐Linking of IκBα Drives NF‐κB Nuclear Translocation to Promote SASP in Senescent Microglia
Source: Aging Cell. 2025 Jan 3;24(5):e14463. doi: 10.1111/acel.14463 (PMC12073898; doi:10.1111/acel.14463)
Supplement: Supplementary file 1 — Data S1. [file ACEL-24-e14463-s001.docx]

**Additional Figures, Tables, and Legends**

**Additional Table 1. Primer information**

| **Gene name** | **Primer sequences** |
| --- | --- |
| **TNFα** | Forward 5`-GGTGCCTATGTCTCAGCCTCTT-3` |
|  | Reverse 5`-GCCATAGAACTGATGAGAGGGAG-3` |
| **IL1α** | Forward 5`-ACGGCTGAGTTTCAGTGAGACC-3` |
|  | Reverse 5`-CACTCTGGTAGGTGTAAGGTGC-3` |
| **IL6** | Forward 5`-TACCACTTCACAAGTCGGAGGC-3` |
| **IL8** | Reverse 5`-CTGCAAGTGCATCATCGTTGTTC-3`  Forward 5`-GGTGATATTCGAGACCATTTACTG-3`  Reverse 5`-GCCAACAGTAGCCTTCACCCAT-3` |
| **MMP3** | Forward 5`-CTCTGGAACCTGAGACATCACC-3` |
|  | Reverse 5`-AGGAGTCCTGAGAGATTTGCGC-3` |
| **MMP12** | Forward 5`-CACACTTCCCAGGAATCAAGCC-3` |
|  | Reverse 5`-TTTGGTGACACGACGGAACAGG-3` |
| **CXCL1** | Forward 5`-TCCAGAGCTTGAAGGTGTTGCC-3` |
|  | Reverse 5`-AACCAAGGGAGCTTCAGGGTCA-3` |
| **CXCL2** | Forward 5`-CATCCAGAGCTTGAGTGTGACG-3` |
|  | Reverse 5`-GGCTTCAGGGTCAAGGCAAACT3` |
| **CXCL10** | Forward 5`-ATCATCCCTGCGAGCCTATCCT-3` |
|  | Reverse 5`-GACCTTTTTTGGCTAAACGCTTTC-3` |
| **P16** | Forward 5`-TGTTGAGGCTAGAGAGGATCTTG-3` |
|  | Reverse 5`-CGAATCTGCACCGTAGTTGAGC-3` |
| **P21** | Forward 5`-TCGCTGTCTTGCACTCTGGTGT-3` |
|  | Reverse 5`-CCAATCTGCGCTTGGAGTGATAG-3` |
| **P53** | Forward 5`-AATTCGAGGCATGGTCCCTGAC-3` |
|  | Reverse 5`-GGTGTTGAAGACAACCTCGGAAG-3` |
| **β-Action** | Forward 5`-CGAGGATGGCTTTCTGGTGA-3` |
|  | Reverse 5`-ATACTTGAGGCGGAGGGTCTG-3` |


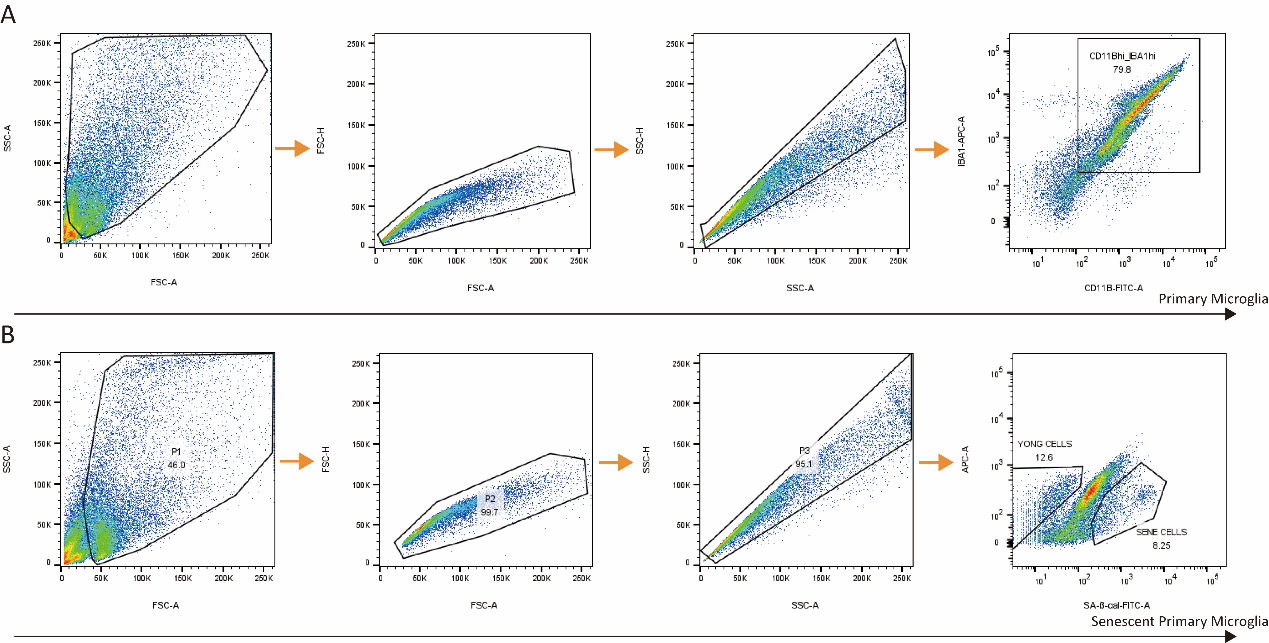


**Additional Figure 1. Primary senescent microglia were sorted and obtained using flow cytometry.**

(A) Flow cytometry was performed to obtain more pure primary microglial cells (CD11b _hi_ and IBA1_hi_).

(B) Senescent microglia were identified and obtained using the SPiDER-βGal assay by flow cytometry.


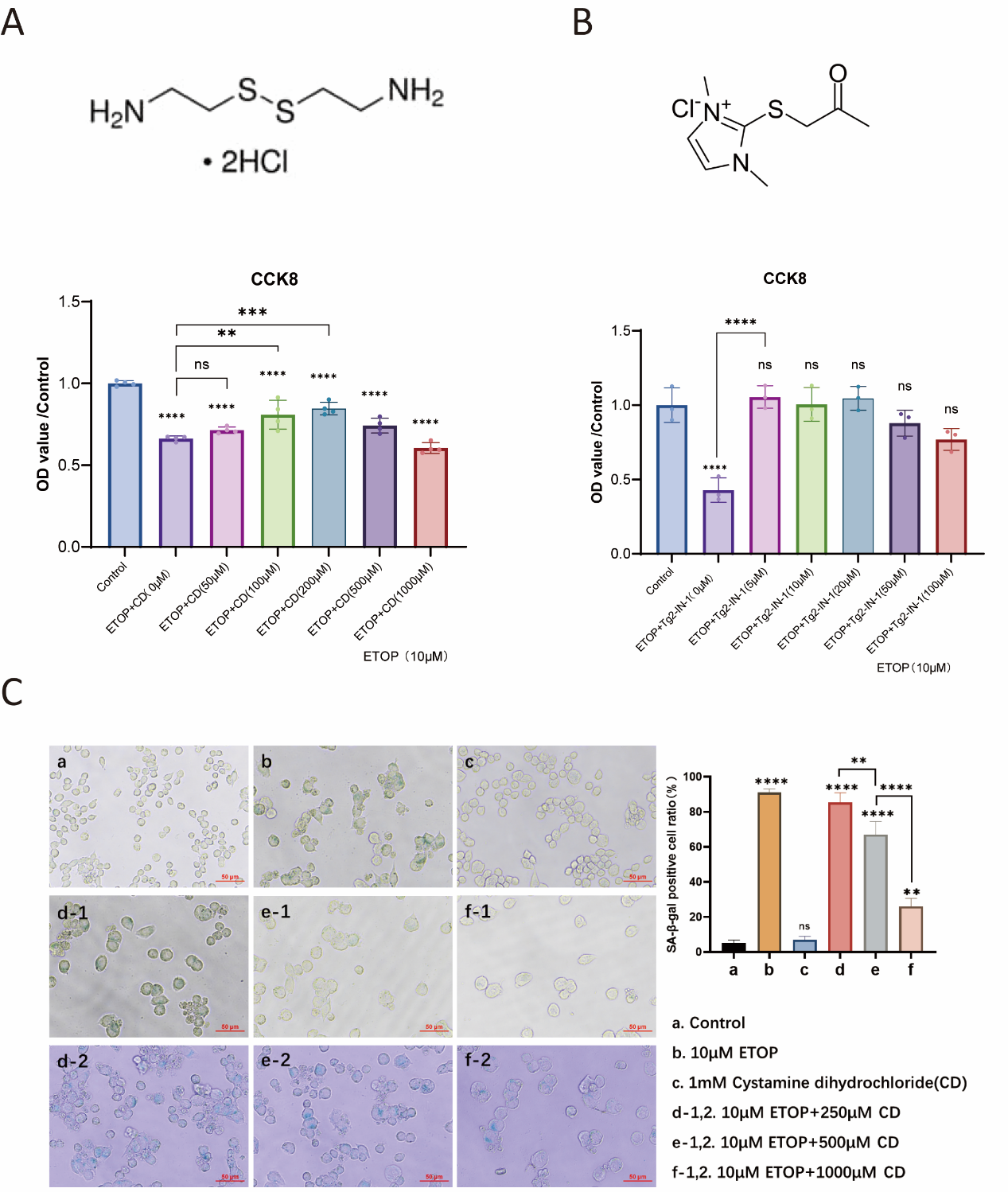


**Additional Figure2. Tgm2 inhibitors slow down cellular senescence.**

(A) Tgm2 inhibitors Cys-D inhibit the growth of senescent microglia, Simultaneous treatment of microglia with different concentrations of Cys-D and 10uM Etoposide.

(B) Tgm2 inhibitors Tg2-IN1 inhibit the growth of senescent microglia, Simultaneous treatment of microglia with different concentrations of Tg2-IN1 and 10uM Etoposide.

(C) SA-β-gal staining of senescent microglia with different concentrations of Cys-D.

Data were analyzed by one-way ANOVA. *P*>0.05 is indicated by ns; *p*<0.01 is indicated by ⁎⁎; *p*<0.001 is indicated by ⁎⁎⁎; *p*<0.0001 is indicated by ⁎⁎⁎⁎.


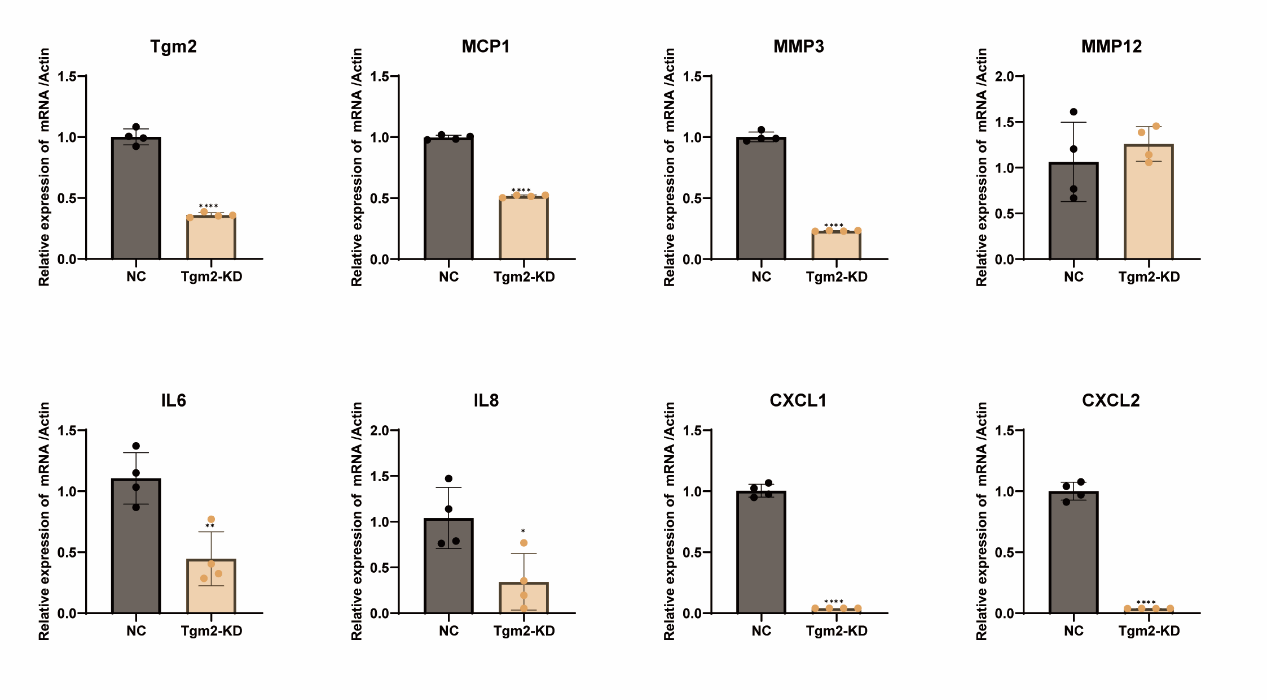


**Additional Figure 3. BV2 cells with Tgm2 knockdown have a lower baseline of inflammatory factors. (n=4)**

Data were analyzed by Student’s *t*-test. *p*<0.05 is indicated by ⁎; *p*<0.01 is indicated by ⁎⁎; *p*<0.0001 is indicated by ⁎⁎⁎⁎.

**
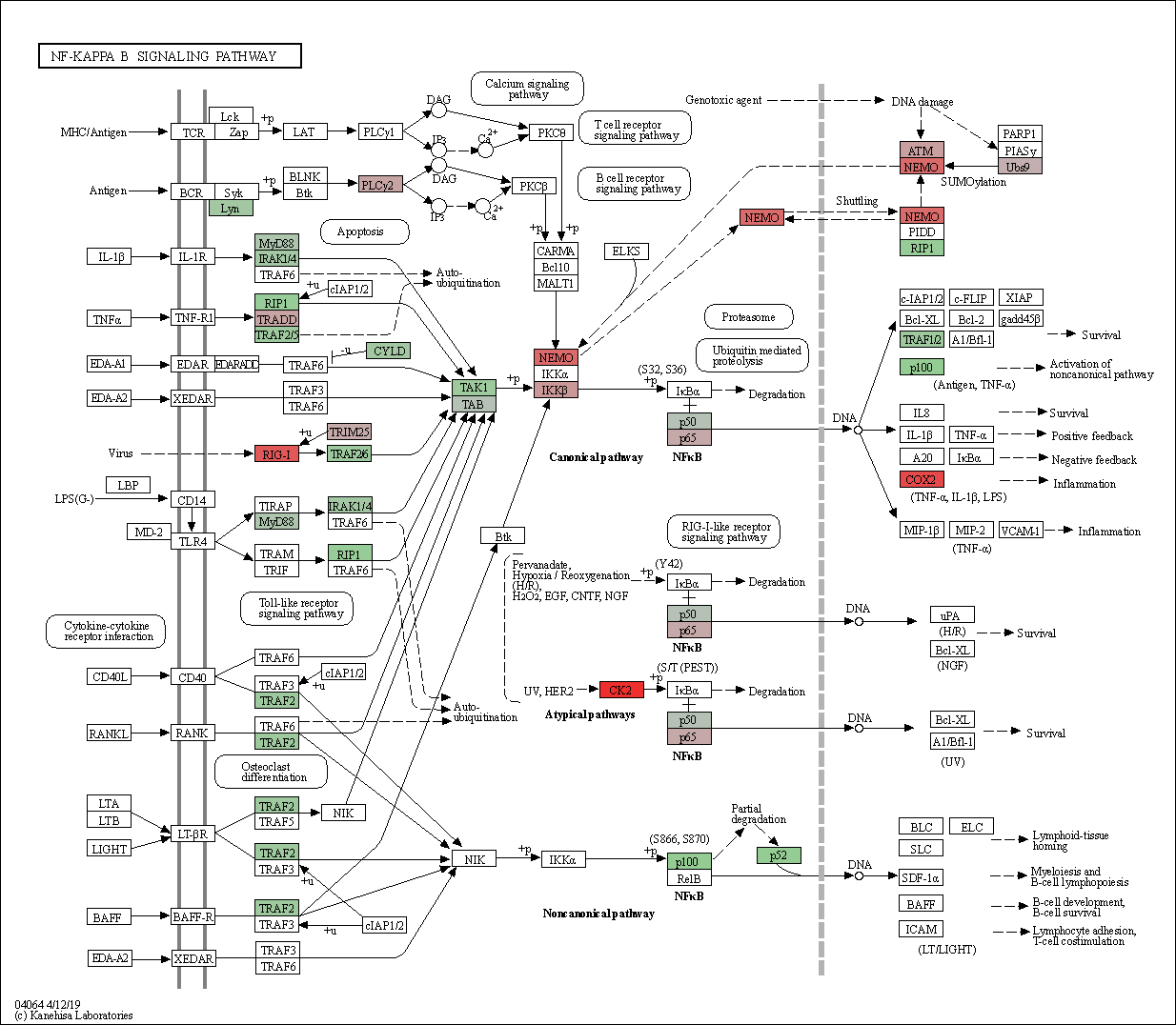
**

**Additional Figure 4. Enrichment analysis of the NF-κB pathway was conducted on the data obtained from TMT quantitative proteomics analysis of senescent microglia cells.**


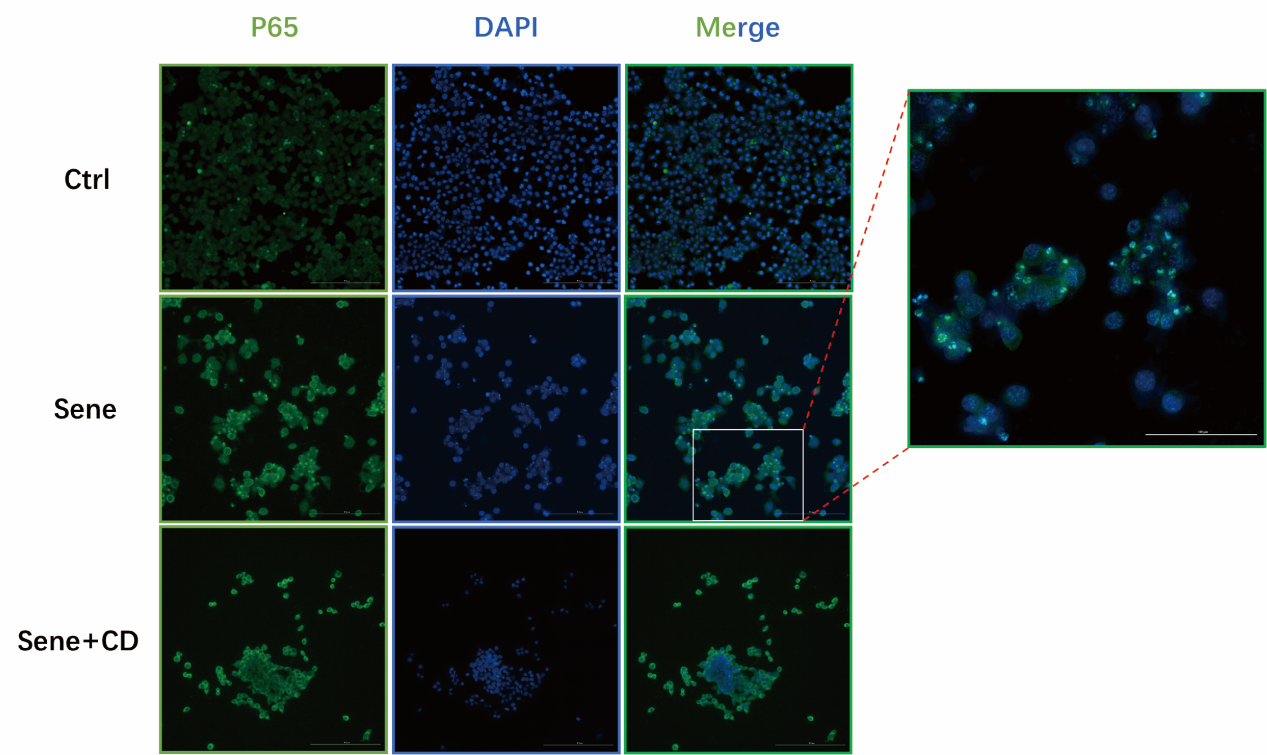


**Additional Figure 5. Tgm2 enzyme inhibitors treatment reduces the P65 in the nucleus of senescent BV2 cells by immunofluorescence.** Scale bar: 100μm.


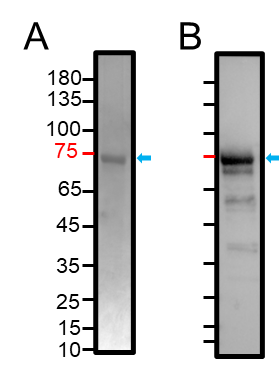


**Additional Figure 6. SDS-PAGE and immunoblot analysis of Tgm2 purified.**

We constructed the Tgm2 plasmid and transfected it into HEK-293F cells, and expressed recombined Tgm2 proteins. From the 100 ml cell, we obtained 1 mg proteins. SDS-PAGE and immunoblot analysis indicated that under the reducing condition Tgm2 had apparent molecular masses of 75 kDa, similar to its calculated masses of 78 kDa (https://web.expasy.org/compute_pi/). Some weak smaller bands showed in the immunoblot result may be due to the protein degradation from the N-terminus.


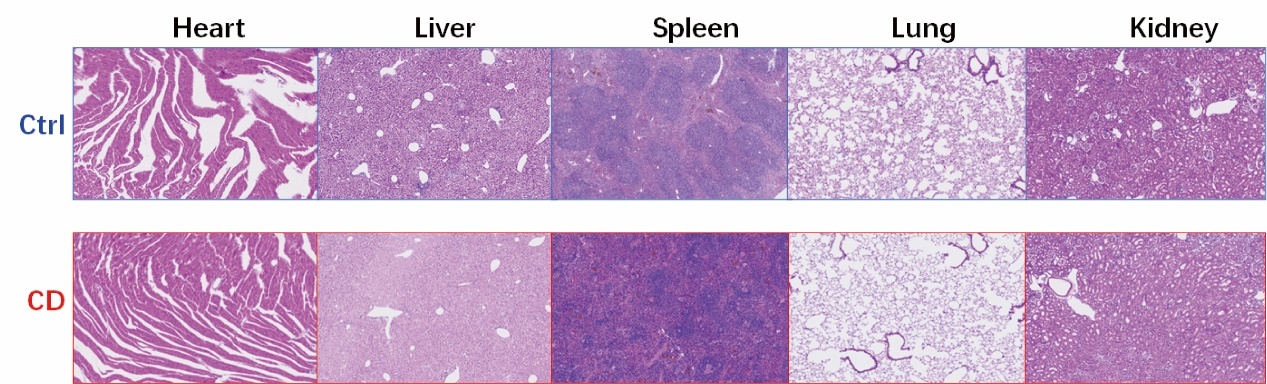


**Additional Figure 7. Histopathological staining results of the main visceral tissues in aged mice orally administered with Cys-D.**
